# The impact of university students' AI attitudes on AI-assisted creativity: the mediating role of AI usage motivation and the moderating role of AI dependency

**DOI:** 10.3389/fpsyg.2026.1735465

**Published:** 2026-03-16

**Authors:** Jianfeng Yin, Tingting Yuan, Yueying Zhang, Guizhen Yang, Xinghua Wang

**Affiliations:** 1 School of Education Science, Qingdao University, Qingdao, China; 2 Boya International College, Yantai Institute of Science and Technology, Yantai, China; 3 Normal College, Ningbo University, Ningbo, China; 4 Faculty of Applied Sciences, Macao Polytechnic University, Macao, China

**Keywords:** AI attitudes, AI dependency, AI-assisted creativity, external regulation, identified regulation, intrinsic motivation

## Abstract

**Introduction:**

With the growing use of artificial intelligence (AI) in higher education, understanding how students develop creativity in AI-assisted learning environments has become increasingly important. This study examines how students' attitudes toward AI influence their AI-assisted creativity, focusing on the mediating role of AI usage motivation and the moderating role of AI dependency. The research is informed by the Theory of Planned Behavior and Self-Determination Theory.

**Methods:**

Data were collected through a questionnaire survey of 347 university students. Structural equation modeling and bootstrap methods were used to test the relationships among AI attitudes, usage motivation (intrinsic motivation, identified regulation, and external regulation), AI dependency, and AI-assisted creativity.

**Results:**

The results show that AI attitudes have a significant positive effect on AI-assisted creativity, and this relationship is fully mediated by the three types of usage motivation. Among them, identified regulation serves as the strongest mediator. The moderating role of AI dependency differs across motivation types. Higher AI dependency weakens the positive effect of intrinsic motivation on creativity but strengthens the effect of external regulation, while the pathway through identified regulation remains largely stable.

**Discussion:**

These findings highlight the importance of motivation internalization in promoting creativity in AI-assisted learning contexts. The results suggest that encouraging more self-endorsed forms of motivation may help students use AI tools more creatively, offering theoretical insights and practical guidance for integrating AI into higher education to support student creativity.

## 1 Introduction

A positive attitude can enhance motivation to engage in creative behavior and cultivate personal creativity before finding creative solutions to complex problems (Basadur and Basadur, 2011; He, 2023). With the rapid adoption of generative artificial intelligence (AI), many university students are now collaborating with AI in creative tasks such as writing and programming. However, attitudes toward such use vary: some students express concern about institutional penalties, while others demonstrate confidence (Baek et al., 2024). Overall, student attitudes toward AI in learning contexts are largely positive, and proactive attitudes, when combined with actual AI use, have been shown to enhance creativity (Katsantonis and Katsantonis, 2024; Habib et al., 2024).

Recent studies indicate a positive, though modest, correlation between creativity and academic performance (Glaveanu et al., 2020). This highlights the importance of establishing creative learning principles and developing pedagogical task systems that incorporate key features of creative problem-solving while enhancing metacognitive engagement (Tin, 2011; Ellis, 2016). However, as more students employ generative AI in academic tasks, its impact on creativity remains uncertain; it may either foster or hinder creative development (Habib et al., 2024). This uncertainty appears linked to the type of student–AI collaboration. For example, Kim et al. (2022) propose a three-phase typology: learning about AI, learning from AI, and learning together.

Despite generally positive attitudes toward AI, students often lack a clear understanding of its appropriate role in learning. There remains limited insight into the nature, impact, and areas for improvement of student–AI collaboration in cooperative learning contexts, as well as the precise role of student–AI synergy in academic tasks (Kim and Cho, 2023). This suggests that while AI can support learning, it may both enhance and diminish creativity depending on how roles are balanced. Clarifying the respective functions of students and AI is therefore essential. Against this backdrop, the present study explores how university students' attitudes toward AI influence creativity within AI-assisted learning contexts.

At the same time, the risks of AI misuse cannot be overlooked. Over-optimism and maladaptive motivations may result in over-reliance and even academic dishonesty (Niloy et al., 2024). Although positive attitudes may encourage AI adoption, they do not guarantee high creativity. Research suggests that heavy reliance on AI may impair cognitive capacities, particularly creative thinking (George et al., 2024; Gerlich, 2025). Some studies even show weak correlations between AI attitudes and creativity (e.g., *R*^2^ = 0.05), suggesting that creativity is more strongly shaped by usage motivations, especially extrinsic motivation, which appears to support creative outcomes (Chen and Zhao, 2025).

Attitudes and motivation are closely interrelated. Previous research shows that perceived ease of use of ChatGPT is not significantly related to behavioral intention, whereas intrinsic motivation is the strongest predictor of use (Lai et al., 2023). Other studies indicate that AI attitudes exert limited influence on learning motivation but are significantly associated with academic performance (Li, 2023; Bation and Pudan, 2024). AI usage motivation ultimately affects behavior through the mediation of attitudes (Kang et al., 2024). Moreover, a paradox has been identified between AI self-efficacy and dependency (Zhang and Xu, 2025). Taken together, these findings imply that while positive attitudes may encourage AI use, they also risk fostering dependency, and maladaptive motivations may undermine creativity, issues warranting serious attention from researchers and educators.

Much prior research has examined how AI attitudes and usage motivations shape behavioral intentions (Kang et al., 2024) and how AI use affects creativity (Habib et al., 2024). Yet, there is still limited understanding of the distinct roles of AI attitudes and qualitatively different types of usage motivation, ranging from autonomous to controlled forms, in shaping student creativity. This gap presents opportunities to investigate how attitudes, motivations, and dependency interact to influence creative outcomes.

Drawing on the Theory of Planned Behavior, students' attitudes toward AI are expected to shape intentions to use it, thereby facilitating collaboration in academic tasks. However, a strong intention to use AI does not necessarily translate into greater creativity. Self-Determination Theory further suggests that motivations differ in their degree of internalization, which leads to distinct effects on creativity (Zhu et al., 2016). Thus, it is not only important to know whether students hold positive attitudes toward AI, but also what motivates them to use it, especially in tackling complex academic challenges, as this interplay critically influences creative development.

Accordingly, this study integrates the Theory of Planned Behavior and Self-Determination Theory to examine whether and how AI attitudes directly foster or indirectly trigger creativity through motivational mechanisms, while also considering the role of AI dependency. These insights are crucial for enhancing students' ability to leverage AI in solving problems creatively. Specifically, the study addresses the following research questions:

- RQ1. How are AI attitudes and AI usage motivation associated with AI-assisted creativity in the context of university students completing academic tasks?
- RQ2. How do different types of AI usage motivation, particularly intrinsic versus controlled forms such as identified and external regulation, differentially affect AI-assisted creativity?
- RQ3. How does AI dependency interact with different types of AI usage motivation to influence AI-assisted creativity?

## 2 Literature review and hypotheses development

### 2.1 The relationship between AI attitude and AI-assisted creativity

University students' attitudes toward AI typically encompass both positive and negative dimensions. Much of the existing research, grounded in the Technology Acceptance Model (TAM), has examined perceived usefulness, perceived ease of use, learning outcomes, perceived barriers, and ethical concerns related to AI (Marengo et al., 2025). According to the Theory of Planned Behavior, attitudes shape intentions, which in turn drive behaviors (Hill et al., 1977). Attitude is often defined as a psychological tendency “expressed by evaluating a particular entity with some degree of favor or disfavor” and consists of affective, behavioral, and cognitive components. Accordingly, students' AI attitudes can be conceptualized as evaluative judgments and intentions across cognitive, affective, and behavioral dimensions.

Creativity has long been recognized as a multidimensional construct. Torrance (1966) identified three core traits (flexibility, fluency, and originality) and developed a creativity index based on them. Guilford (1967) argued that intelligence tests failed to capture originality or innovation, reinforcing the view that creativity transcends traditional notions of intelligence, especially in addressing complex problems. These dimensions of creativity are widely applied in educational an design contexts.

With the growing use of AI in creative domains, university students increasingly employ AI to complete challenging learning tasks. In these contexts, AI becomes embedded in human creative processes, often blurring the boundaries between student and AI contributions and creating potential imbalances. To capture this phenomenon, we introduce the concept of AI-assisted creativity, which reflects the synergy between human and AI inputs in learning tasks. Because it is difficult to attribute creative outcomes exclusively to either the student or the AI, this study explores the relationship between students' AI attitudes and AI-assisted creativity.

AI attitude is closely tied to behavioral intentions to use AI and may also influence tendencies toward creative problem-solving. For example, affective and cognitive attitudes toward AI have been shown to predict AI awareness and usage, whereas behavioral attitudes do not (Otermans et al., 2025). In terms of the attitude–creativity link, some scholars argue that creativity itself can be viewed as an attitude, with positive adjustments enhancing creative performance and negative ones constraining it (Brennan and Dooley, 2005). Empirical findings further suggest that student–AI collaboration in tasks such as drawing significantly affects the creativity of outcomes, depending on students' AI attitudes (Kim and Lee, 2023). Similarly, when AI is perceived as human-like, collaboration leads to higher creative fluency and greater trust in AI, which strengthens collaborative creativity (Hwang and Won, 2021). AI attitudes have also been shown to positively influence creative engagement (Korayim et al., 2025).

Therefore, in the context of AI-assisted learning tasks, we hypothesize:

H1. AI attitude has a significant positive effect on AI-assisted creativity.

### 2.2 The relationship between AI attitude and AI usage motivation

The misuse of AI in higher education may undermine students' intrinsic learning motivation, leading to academic dishonesty and superficial learning. It is therefore critical to balance technological advancement with the preservation of intrinsic motivation (Alasgarova and Rzayev, 2024). In this study, we distinguish between learning motivation and AI usage motivation, the latter being particularly relevant in the context of AI-assisted tasks.

Drawing on Self-Determination Theory (Ryan and Deci, 2000a,b), motivation is conceptualized along a continuum from non-autonomous to autonomous, including intrinsic motivation, integrated regulation, identified regulation, introjected regulation, external regulation, and amotivation. We focus on three representative dimensions: Intrinsic motivation (most autonomous), Identified regulation (internalized extrinsic motivation reflecting recognition of AI's value), and External regulation (most controlled, driven by external pressures or rewards).

Attitude and motivation are closely interrelated, and this relationship extends to AI contexts. For instance, AI usage motivation is significantly correlated with AI attitude (Yurt and Kaşarci, 2024). Motivation can positively influence behavioral intention through the mediation of AI acceptance attitudes (Kang et al., 2024). In the case of voice AI assistants, informational motivation predicts both usage attitude and behavioral intention (Choi and Drumwright, 2021). Conversely, positive AI attitudes can foster motivation by increasing interest and engagement with AI (Bewersdorff et al., 2025).

Research also indicates that attitudes and motivation jointly promote creativity. For example, employees' positive attitudes enhance workplace creativity through the mediation of work motivation (Saintilan and Schreiber, 2023), while stronger intrinsic motivation amplifies the role of servant attitudes in fostering creativity (Ruiz-Palomino and Zoghbi-Manrique-de-Lara, 2020). Similarly, in the AI-assisted learning context, different dimensions of AI usage motivation may mediate the link between AI attitude and creativity.

Thus, we propose the following hypotheses:

H2. Intrinsic motivation in AI usage mediates the relationship between AI attitude and AI-assisted creativity.

H3. Identified regulation in AI usage mediates the relationship between AI attitude and AI-assisted creativity.

H4. External regulation in AI usage mediates the relationship between AI attitude and AI-assisted creativity.

### 2.3 The relationship between AI usage motivation and AI-assisted creativity

Human creativity is strongly associated with intrinsic motivation (IM). While extrinsic motivation, driven by external incentives, may sometimes stimulate creativity, it also carries the risk of undermining it (Li et al., 2025; Schmidhuber, 2010). Intrinsic motivation supports process-oriented creativity, whereas extrinsic motivation tends to produce outcome-oriented effects (Forgeard and Mecklenburg, 2013). In higher education, intrinsic motivation mediates the relationship between self-efficacy and creativity, while extrinsic motivation often moderates it (Prabhu et al., 2008). In art education, extrinsic rewards may inhibit creativity, as children's artistic expression often emerges from intrinsic interest (Jaquith, 2011).

This complexity also applies in AI-assisted contexts. For example, intrinsic motivation, particularly play-based engagement, enhances computer-mediated creativity in young learners (Parwoto et al., 2024). By contrast, most computational creativity systems are driven by extrinsic motives (Zhang Q. et al., 2024). Nevertheless, intrinsic motivation remains crucial in human–AI collaborative creativity (Moura, 2023). Effective integration of AI in education thus requires a nuanced understanding of students' motivations (An et al., 2025). Adler and Chen (2011) further argue that large-scale collaborative creativity (LSCC) relies on both intrinsic and identified motivation, supported by internalized values.

Accordingly, we hypothesize:

H5. Intrinsic motivation in AI usage has a significant positive effect on AI-assisted creativity.

H6. Identified regulation in AI usage has a significant positive effect on AI-assisted creativity.

H7. External regulation in AI usage has a significant positive effect on AI-assisted creativity.

### 2.4 The moderating role of AI dependency

AI dependency is not a clinical disorder but rather a behavioral pattern characterized by excessive reliance on AI due to overtrust in its capabilities (Zhai et al., 2024). Studies in higher education have linked AI dependency to academic dishonesty, reduced social engagement, and homogenized outputs (Crawford et al., 2024; Tilawat, 2025). Overreliance may also weaken cognitive skills, learning motivation, problem-solving, critical thinking, and creativity (Morales-García et al., 2024; Zhang S. et al., 2024; Ahmad et al., 2023; Zhang and Xu, 2025).

That said, appropriate use of AI, under conditions of sufficient engagement and AI literacy, can enhance creativity. The concern lies in excessive dependency, which is likely to undermine it.

Therefore, we hypothesize:

H8. AI dependency moderates the mediating effect of intrinsic motivation in AI usage.

H9. AI dependency moderates the mediating effect of identified regulation in AI usage.

H10. AI dependency moderates the mediating effect of external regulation in AI usage.

**Figure 1:**
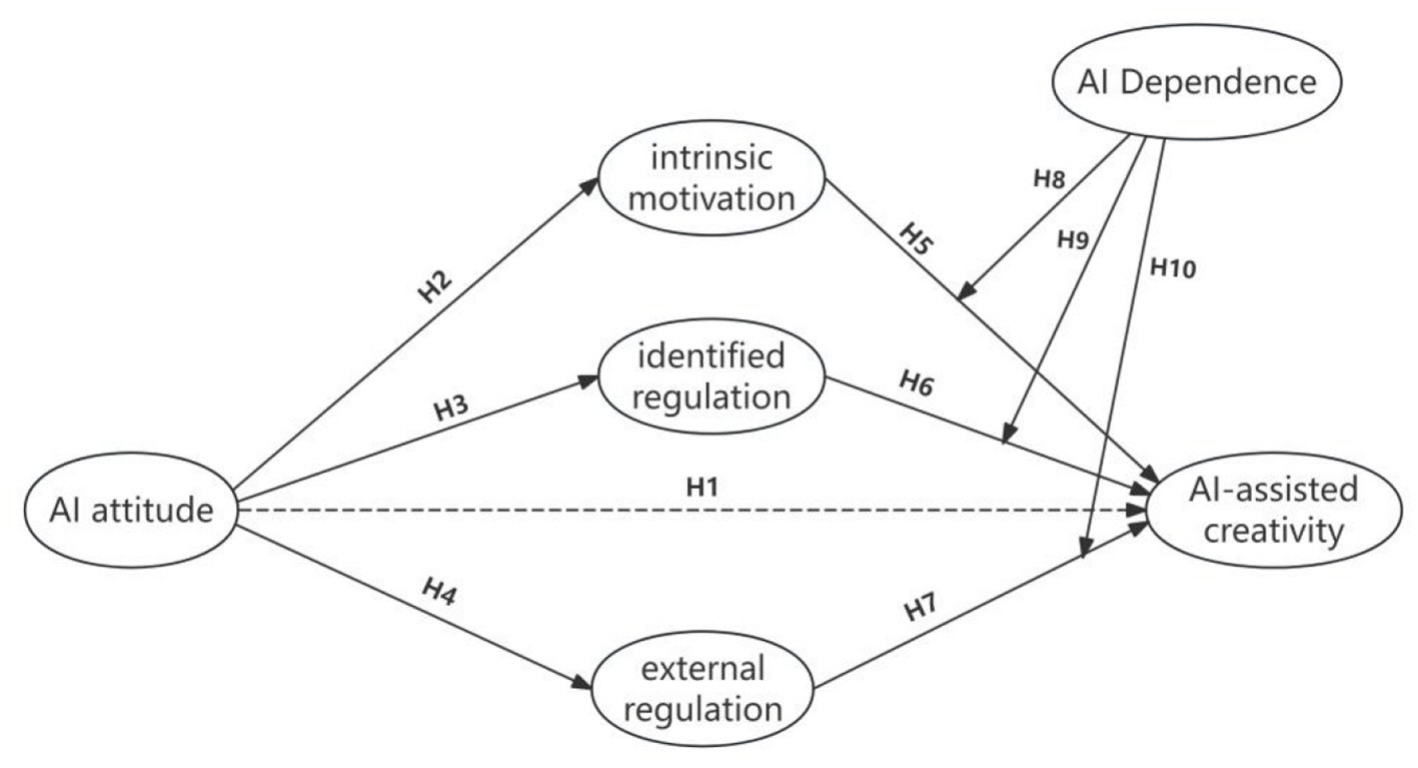
Hypothesized model.

Finally, this study developed a theoretical model illustrating how AI attitudes influence AI-assisted creativity, as shown in Figure 1.

## 3 Research design

### 3.1 Questionnaire design

#### 3.1.1 AI usage motivation

Based on Self-Determination Theory (SDT) and classical definition of intrinsic and extrinsic motivation (Ryan and Deci, 2000a,b), this study focused on three of the six motivational dimensions: intrinsic motivation, identified regulation, and external regulation. Nine items measuring AI usage motivation were adapted from the Academic Motivation Scale validated by Barkoukis et al. (2008) and contextualized to the setting of Chinese university students using AI to complete learning tasks. Intrinsic motivation reflects engagement driven by inherent interest and enjoyment, as illustrated by items such as “I use AI learning tools because I find the learning process itself interesting,” which capture the interest-driven and autonomy-oriented nature of SDT. Identified regulation represents a relatively autonomous form of extrinsic motivation, arising from the personal endorsement of a behavior's value, with items emphasizing recognition of academic value and awareness of resource utilization. External regulation, by contrast, is characterized by motivation driven by external factors, reflected in items such as “I use AI learning tools because I want to achieve good grades in courses” and “I use AI learning tools because the university encourages us to use them,” which align with incentive-driven and compliance-based aspects of SDT.

#### 3.1.2 AI attitude

Students' attitudes toward AI were assessed with reference to the University Students' AI Attitude Scale developed and validated by Katsantonis and Katsantonis (2024). The original instrument contains 25 items spanning cognitive, affective, and behavioral dimensions, from which four items were carefully selected and adapted to match the present research context. The cognitive dimension was captured through items such as “How important do you think AI tools are in higher education?” The affective dimension incorporated peer-comparison elements, for example, “Do you approve of other students using AI tools?” reflecting the influence of peer norms and social acceptance. The behavioral dimension emphasized active engagement, as in the item “When using AI tools, do you try different functions to complete tasks?” which signals students' willingness to explore AI-assisted learning.

#### 3.1.3 AI-assisted creativity

AI-assisted creativity was measured using items adapted from the Torrance Tests of Creative Thinking (TTCT; Torrance, 1966), which conceptualize creativity through fluency, flexibility, and originality. To fit the context of human–AI collaboration in learning, the items were reformulated to emphasize generative AI applications. Examples include “I can often use generative AI to generate a large number of ideas and solutions,” “When using generative AI, I can explore solutions to problems from multiple perspectives,” and “I can often guide generative AI to help me devise novel problem-solving approaches.” A total of six items were used to capture the extent to which students engage in collaborative creativity with AI tools.

#### 3.1.4 AI dependency

AI dependency was assessed through scales adapted from Andreassen et al. (2016) and subsequently modified for AI contexts by Zhai et al. (2024) and Zhang S. et al. (2024), along with the University Students' AI Dependency Scale developed by Morales-García et al. (2024). Five items were integrated and contextualized to evaluate dependency in AI-assisted learning. Illustrative items include “I feel less confident completing academic tasks without AI assistance,” which reflects habitual and psychological dependency, and “After using AI, I find myself engaging less in deep reading and thinking,” which highlights cognitive substitution and reduced autonomy. This construct thus captures the potential risks associated with overreliance on AI, including diminished cognitive engagement and academic self-efficacy.

### 3.2 Participants and procedure

The data for this study were collected from undergraduate students at a comprehensive university in China, with the aim of investigating the factors that influence AI-assisted creativity in learning contexts. A standardized questionnaire was administered, and to ensure participants had actual experience of using AI in learning tasks, a screening question was included at the beginning of the survey. Only respondents who confirmed prior AI use in learning contexts were able to proceed. During data cleaning, invalid responses—such as patterned answering or inconsistencies between the screening question and subsequent items—were excluded. After this process, 347 valid questionnaires were retained for analysis. The demographic characteristics of the final sample are summarized in Table 1.

**Table 1:** Basic information of samples (*N* = 347).

**Variable name**	**Category**	**Count**	**Percentage (%)**
Gender	Male	47	13.5
	Female	300	86.5
Academic year	Freshman	139	40.1
	Sophomore	71	20.5
	Junior	72	20.7
	Senior	56	16.1
	Graduate	9	2.6
AIGC usage frequency	0–2 times/month	91	26.2
	3–5 times/month	132	38.0
	6–10 times/month	71	20.5
	>10 times/month	53	15.3

### 3.3 Data analysis methods

Data analysis was performed using SPSS 26.0 and AMOS 24. First, item analysis, exploratory factor analysis, and internal consistency reliability tests were conducted to evaluate the reliability and validity of the scales measuring AI attitude, AI usage motivation, and AI-assisted creativity, ensuring the measurement tools were both reliable and valid. Second, descriptive statistical analysis was carried out on the basic demographic variables to illustrate the overall distribution and fundamental characteristics of the sample. Third, Pearson correlation analysis was employed to examine the relationships among the key variables—AI attitude, AI usage motivation, and AI-assisted creativity—laying the groundwork for subsequent hypothesis testing. A structural equation model was constructed in AMOS to further examine the mediating role of AI usage motivation in the relationship between AI attitude and AI-assisted creativity. On this basis, the PROCESS macro was used with the Bootstrap method to test the moderating effect of AI dependency within the mediation model.

## 4 Empirical analysis

### 4.1 Descriptive statistics and correlation analysis

The means, standard deviations, and correlation coefficients of the variables are presented in Table 2. The mean score for AI attitude was above the midpoint, indicating that university students generally hold a positive inclination toward the application of AI technology in learning contexts. As an emerging technological tool, AI has gained a certain level of acceptance among students. Meanwhile, the mean score for identified regulation (*M* = 3.97) was higher than those for intrinsic motivation (*M* = 3.60) and external regulation (*M* = 3.41), suggesting that students rely more heavily on identified regulation in their use of AI. The mean intrinsic motivation score (*M* = 3.60) was higher than that of external regulation (*M* = 3.41), indicating that students are driven more by personal interest or internal satisfaction than by external rewards or pressures when using AI tools. Furthermore, the mean score for AI-assisted creativity was 3.94, reflecting a relatively high level of creative performance in human–AI collaboration.

**Table 2:** Descriptive statistics and correlation coefficients of variables (*N* = 347).

**Variable**	**M ±SD**	**AI attitude**	**Intrinsic motivation**	**Identified regulation**	**External regulation**	**AI-assisted creativity**
AI attitude	3.85 ± 0.69	1				
Intrinsic motivation	3.60 ± 0.85	0.315^**^	1			
Identified regulation	3.97 ± 0.72	0.527^**^	0.555^**^	1		
External regulation	3.41 ± 0.80	0.383^**^	0.351^**^	0.479^**^	1	
AI-assisted creativity	3.94 ± 0.69	0.437^**^	0.509^**^	0.636^**^	0.499^**^	1

^**^Indicates that the correlation is significant at the 0.01 level (two-tailed).

Correlation analysis, conducted using Pearson correlation coefficients, revealed significant pairwise correlations among all variables (*p* < 0.01), providing preliminary support for the hypothesized relationships. Specifically, AI attitude was significantly and positively correlated with intrinsic motivation, identified regulation, and external regulation, reflecting its broad influence across different motivational types. In addition, AI attitude was significantly and positively correlated with AI-assisted creativity, offering initial evidence of the positive role of a positive attitude in fostering creative outcomes. Intrinsic motivation, identified regulation, and external regulation were also significantly and positively correlated with AI-assisted creativity, with identified regulation showing the strongest correlation with creativity, highlighting its central role in driving collaborative human–AI creativity. The correlation analysis preliminarily revealed consistent patterns among the variables, providing a empirical foundation for further analysis.

### 4.2 Reliability and validity analysis

Reliability analysis was conducted to assess the stability and consistency of the measurement scales. Cronbach's α coefficient was used as an indicator of internal consistency reliability. The results showed that all variables had Cronbach's α values exceeding 0.8, indicating strong internal consistency among the items within each dimension and confirming that the measurements were stable and reliable.

Exploratory factor analysis was performed, and Harman's single-factor test revealed five factors with eigenvalues greater than 1, which collectively accounted for 70.892% of the total variance. The first factor explained 20.155% of the variance, which was less than half of the total, suggesting that common method bias was not a serious concern in this study.

Validity was assessed through convergent validity and model fit indices. Convergent validity, which reflects the extent to which items of the same construct converge, was evaluated using average variance extracted (AVE) and composite reliability (CR). As shown in Table 3, all AVE values exceeded 0.5, indicating that the items sufficiently captured the variance of their respective constructs. All CR values were above 0.8, surpassing the threshold of 0.7, further supporting the internal consistency and convergent validity of the scales.

**Table 3:** Reliability and validity analysis.

**Variable**	**Latent variable code**	**Standardized loading**	**SMC**	**Cronbach's α**	**CR**	**AVE**
AI attitude	A1	0.794	0.631	0.849	0.849	0.586
	A2	0.719	0.517			
	A5	0.809	0.654			
	A9	0.735	0.54			
Intrinsic motivation	C4	0.767	0.588	0.810	0.804	0.578
	C5	0.79	0.624			
	C6	0.723	0.523			
Identified regulation	C8	0.801	0.641	0.821	0.822	0.606
	C9	0.728	0.53			
	C10	0.805	0.648			
External regulation	C1	0.72	0.518	0.814	0.822	0.607
	C2	0.851	0.724			
	C3	0.762	0.58			
AI-assisted creativity	B1	0.745	0.555	0.886	0.879	0.550
	B2	0.752	0.566			
	B3	0.761	0.579			
	B4	0.67	0.448			
	B5	0.84	0.705			
	B7	0.667	0.445			

Additionally, the model fit indices (details in Table 4) indicated an acceptable fit: χ^2^/*df* = 3.366, which falls within the acceptable range of 1–5; RMSEA = 0.083, indicating acceptable model fit; and goodness-of-fit indices including GFI = 0.876, CFI = 0.907, TLI = 0.888, and IFI = 0.908, all exceeding the benchmark of 0.8, suggesting a satisfactory overall fit between the model and the observed data.

**Table 4:** Model fit indices.

**Commonly used indices**	**Reference criteria**	**Value**
χ^2^/*df*	1–3 Excellent, 3–5 Acceptable	3.366
RMSEA	< 0.08 Excellent, < 0.1 Acceptable	0.083
GFI	>0.9 Excellent, >0.8 Acceptable	0.876
CFI	>0.9 Excellent, >0.8 Acceptable	0.907
TLI	>0.9 Excellent, >0.8 Acceptable	0.888
IFI	>0.9 Excellent, >0.8 Acceptable	0.908

In summary, the reliability and validity analyses demonstrate that the scales used in this study possess good internal consistency and convergent validity, and the model exhibits acceptable fit. This provides a robust measurement foundation for subsequent analysis of the mechanism through which AI usage motivation influences AI-assisted creativity.

### 4.3 Testing the direct effect of AI attitude on AI-assisted creativity

As shown in Table 5, a linear regression analysis was conducted to examine the direct relationship between the independent variable and the dependent variable, without considering mediating or moderating variables. The coefficient analysis revealed a Beta value of 0.437 with *p* < 0.001, indicating that AI attitude had a statistically significant positive influence on students' AI-assisted creativity. Tolerance and variance inflation factor (VIF) values were used to assess multicollinearity. Generally, tolerance greater than 0.1 and VIF less than 10 indicate the absence of severe multicollinearity. The results showed that both tolerance and VIF values for all variables fell within acceptable thresholds, confirming that no serious multicollinearity issues existed among the independent variables. Thus, Hypothesis H1 was supported.

**Table 5:** Results of the direct effect tests.

**Variable**	**Unstandardized coefficients**		**Standardized coefficients**			**Collinearity statistics**	
	**Beta**	**Std. error**	**Beta**	***t***	***p*** **-value**	**Tolerance**	**VIF**
(Constant)	2.266	0.189		12.000	0.000		
AI attitude	0.436	0.048	0.437	9.027	0.000	1.000	1.000

### 4.4 Testing the mediating role of usage motivation

The model was estimated using the maximum likelihood method, and a bias-corrected non-parametric percentile Bootstrap approach with 2,000 resamples was employed to calculate 95% confidence intervals. The path analysis results of the mediation model are presented in Table 6.

**Table 6:** Results of mediation model path analysis.

**Path**	**Estimate**	**S.E**.	**C.R**.	***P***
AI attitude → Intrinsic motivation	0.459	0.067	6.892	0.000
AI attitude → Identified regulation	0.695	0.068	10.278	0.000
AI attitude → External regulation	0.608	0.076	7.953	0.000
Intrinsic motivation → AI-assisted creativity	0.249	0.057	4.349	0.000
Identified regulation → AI-assisted creativity	0.519	0.086	6.052	0.000
External regulation → AI-assisted creativity	0.255	0.053	4.840	0.000
AI attitude → AI-assisted creativity	−0.060	0.092	−0.653	0.513

The results indicate that AI attitude had a significant positive effect on students' intrinsic motivation, identified regulation, and external regulation in AI usage. Among these three dimensions of motivation, AI attitude exerted the strongest influence on identified regulation (β = 0.695), followed by external regulation (β = 0.608). Furthermore, intrinsic motivation, identified regulation, and external regulation were all significantly and positively correlated with AI-assisted creativity, with standardized path coefficients of 0.249, 0.519, and 0.255, respectively. These findings confirm that all three types of motivation positively predict AI-assisted creativity, supporting Hypotheses H5, H6, and H7.

After including the three mediating variables—intrinsic motivation, identified regulation, and external regulation—the direct effect of AI attitude on AI-assisted creativity decreased from 0.437 (*p* < 0.001) to −0.060 (*p* > 0.05). This suggests that, in the presence of these mediators, AI attitude no longer exerts a significant direct effect on AI-assisted creativity. Therefore, AI usage motivation not only mediates this relationship but also exhibits a full mediation effect.

The path test results of the mediation effects and their respective proportions are shown in Tables 7, 8. The total indirect effect was 0.574. The specific indirect effects through intrinsic motivation, identified regulation, and external regulation were 0.08, 0.365, and 0.129, respectively, and their 95% confidence intervals did not include zero, confirming the significance of the mediation effects. Thus, Hypotheses H2, H3, and H4 were supported.

**Table 7:** Results of mediation effect analysis.

**Mediation path**	**Point estimate**	**Indirect effect**		**Bootstrapped 95% CI**			
		**SE**	***Z***	**Bias-corrected**		**Percentile**	
				**Lower**	**Upper**	**Lower**	**Upper**
AI attitude → Intrinsic motivation → AI-assisted creativity	0.080	0.029	2.77	0.007	0.158	0.008	0.160
AI attitude → Identified regulation → AI-assisted creativity	0.365	0.074	4.93	0.225	0.526	0.228	0.530
AI attitude → External regulation → AI-assisted creativity	0.129	0.030	4.30	0.098	0.218	0.100	0.220
Total effect	0.534	0.065	8.22	0.370	0.575	0.375	0.580
Total indirect effect	0.574	0.120	4.79	0.370	0.575	0.375	0.580

**Table 8:** Proportion of mediation effects.

**Mediation path**	Effect size (a^*^b)	**Proportion of total effect (%)**	**Conclusion**
AI attitude → Intrinsic motivation → AI-assisted creativity	0.080 (0.509 × 0.158)	15.0	Full mediation
AI attitude → Identified regulation → AI-assisted creativity	0.365 (0.694 × 0.526)	68.4	
AI attitude → External regulation → AI-assisted creativity	0.129 (0.595 × 0.218)	24.2	

Moreover, the proportion of mediation effects revealed that identified regulation accounted for nearly 70% of the total mediation effect, while the other two motivations contributed relatively less. This indicates that, compared to intrinsic motivation and external regulation, identified regulation provides a stronger explanatory mechanism for the relationship between AI attitude and AI-assisted creativity, serving as the dominant mediating factor in fostering AI-assisted creativity.

### 4.5 Testing the moderating role of AI dependency

This study employed the SPSS PROCESS macro (Model 14) to examine the moderating effect of AI dependency on the three mediating pathways—intrinsic motivation, identified regulation, and external regulation. Detailed results are presented in Tables 9, 10.

**Table 9:** Results of moderation analysis.

**Moderation path**	**AI-assisted creativity**	**Intrinsic motivation**	**Identified regulation**	**External regulation**
Constant	0.895 (1.480)	2.097^**^ (8.274)	1.730^**^ (9.109)	1.765^**^ (7.590)
AI attitude	0.143^**^ (3.089)	0.388^**^ (6.022)	0.577^**^ (11.945)	0.425^**^ (7.195)
AI dependence	0.075 (0.413)			
Intrinsic motivation	0.829^**^ (7.217)			
Intrinsic motivation × AI dependence	−0.208^**^ (−6.041)			
Identified regulation	−0.034 (−0.226)			
Identified regulation × AI dependence	0.118^**^ (2.597)			
External regulation	−0.196 (−1.658)			
External regulation × AI dependence	0.088^*^ (2.357)			
*Sample size (N)*	347	347	347	347
*R* ^2^	0.539	0.095	0.293	0.130
*AdjustedR* ^2^	0.527	0.090	0.288	0.125
*F-statistic*	*F*_(8, 338)_ = 49.428, *p* = 0.000	*F*_(1, 345)_ = 36.264, *p* = 0.000	*F*_(1, 345)_ = 142.693, *p* = 0.000	*F*_(1, 345)_ = 51.772, *p* = 0.000

Values in parentheses represent t-values.

^*^p < 0.05, ^**^p < 0.01.

**Table 10:** Conditional indirect effects.

**Mediator**	**Level**	**Value**	**Effect**	**BootSE**	**BootLLCI**	**BootULCI**
Intrinsic motivation	Low (−1SD)	2.238	0.141	0.040	0.056	0.208
	Mean	3.136	0.068	0.023	0.021	0.112
	High (+1SD)	4.034	−0.004	0.023	−0.050	0.042
Identified regulation	Low (−1SD)	2.238	0.132	0.063	0.038	0.281
	Mean	3.136	0.193	0.042	0.123	0.288
	High (+1SD)	4.034	0.255	0.049	0.165	0.356
External regulation	Low (−1SD)	2.238	0.001	0.024	−0.051	0.044
	Mean	3.136	0.034	0.019	−0.004	0.071
	High (+1SD)	4.034	0.068	0.025	0.022	0.118

First, regarding the moderating effect of AI dependency on the intrinsic motivation pathway, the interaction term between intrinsic motivation and AI dependency had a significant effect on AI-assisted creativity (β = −0.208, *t* = −6.041, *p* < 0.01), indicating that AI dependency significantly moderates the relationship between intrinsic motivation and collaborative creativity. Further conditional indirect effect analysis revealed that when AI dependency was at a low level, the mediating effect of intrinsic motivation was significant, with a 95% confidence interval excluding zero. At the mean level of AI dependency, the mediating effect remained significant but weakened in strength. When AI dependency was high, the mediating effect became non-significant, as the 95% confidence interval included zero. The index of moderated mediation had a 95% confidence interval excluding zero, further confirming the moderating role of AI dependency on the intrinsic motivation pathway. Specifically, as the level of AI dependency increases, the positive mediating effect of intrinsic motivation on AI-assisted creativity gradually weakens and eventually disappears. Thus, Hypothesis H8 was supported.

Second, for the moderating effect of AI dependency on the identified regulation pathway, the interaction term between identified regulation and AI dependency had a significant effect on AI-assisted creativity (β = 0.118, *t* = 2.597, *p* < 0.01). However, conditional indirect effect analysis showed that the mediating effect of identified regulation remained significant whether AI dependency was at a low, mean, or high level, with all 95% confidence intervals excluding zero. The index of moderated mediation had a 95% confidence interval including zero, indicating that the moderating effect of AI dependency on the identified regulation pathway was not significant. In other words, the mediating effect of identified regulation is not influenced by the level of AI dependency. Therefore, Hypothesis H9 was not supported.

Finally, concerning the moderating effect of AI dependency on the external regulation pathway, the interaction term between external regulation and AI dependency had a significant effect on AI-assisted creativity (β = 0.088, *t* = 2.357, *p* < 0.05). Conditional indirect effect analysis indicated that when AI dependency was low, the mediating effect of external regulation was not significant. At the mean level of AI dependency, the mediating effect remained non-significant. However, when AI dependency was high, the mediating effect of external regulation became significant, with a 95% confidence interval excluding zero. The index of moderated mediation had a 95% confidence interval excluding zero, confirming the moderating role of AI dependency on the external regulation pathway. Specifically, as the level of AI dependency increases, the positive mediating effect of external regulation on AI-assisted creativity strengthens, emerging from an initially non-significant state. Thus, Hypothesis H10 was supported.

**Figure 2:**
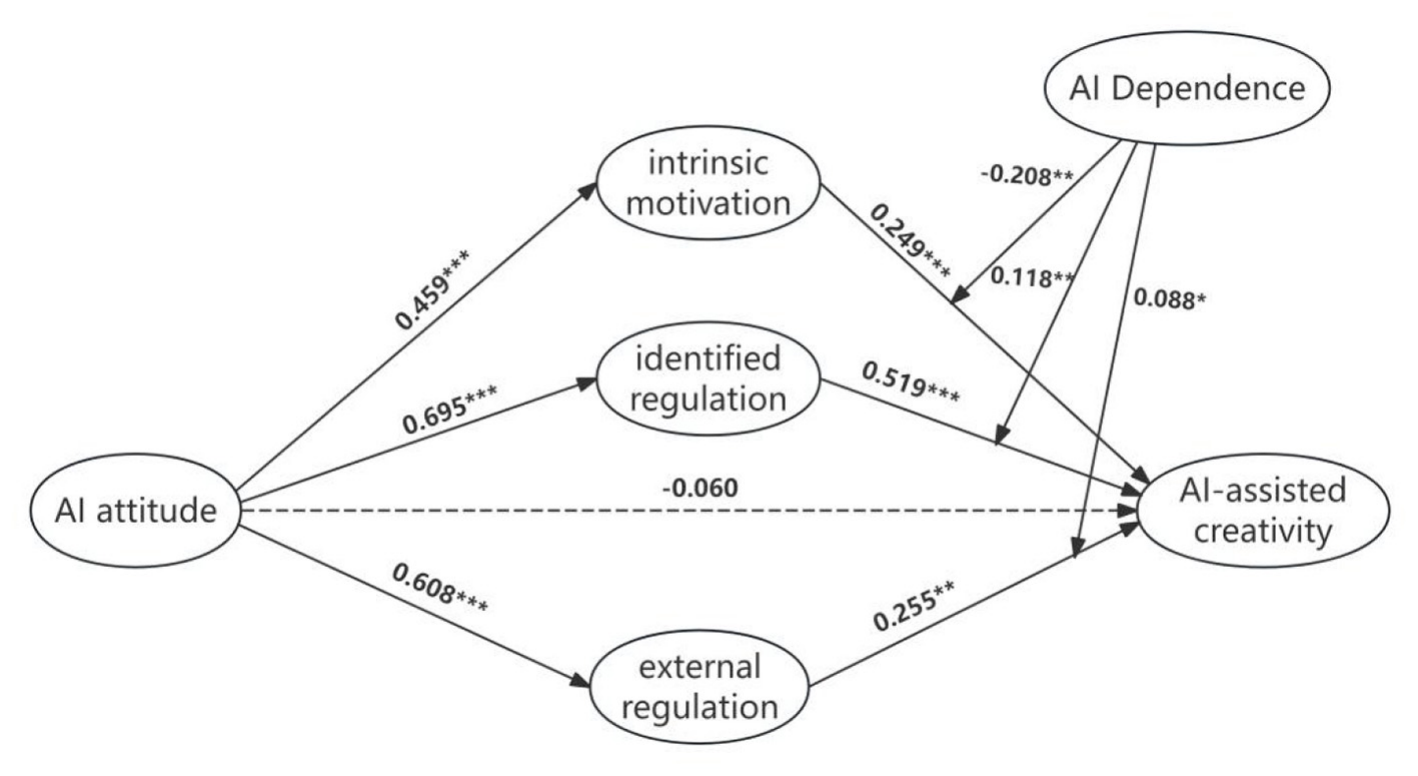
The moderated mediation model. *p < 0.05, **p < 0.01, ***p < 0.001.

The final structural model is presented in Figure 2.

## 5 Discussion

This study aimed to explore university students' attitudes toward using AI for completing learning tasks, along with their different types of motivations, and to analyze the differential impact of these factors on AI-assisted creativity based on Self-Determination Theory and the Theory of Planned Behavior. The creative outputs generated by university students with AI assistance exhibit a human-AI hybrid nature. However, existing research has rarely delved deeply into this form of creativity stimulated by human-AI collaboration. Through an empirical analysis of 347 university students, this study revealed several key findings.

First, this study found that students' attitudes toward AI have a significant positive direct effect on AI-assisted creativity, which aligns with the findings of Korayim et al. (2025). That is, an individual's positive attitude toward technology forms the foundation for its creative use. According to the data, university students generally hold the fundamental belief that “AI can assist learning.” This attitude provides a prerequisite for the subsequent activation of motivation and creative output.

However, the direct effect of AI attitude on AI-assisted creativity completely disappeared after introducing motivation variables, indicating that attitude influences creativity indirectly through motivation. This result differs from the pathway of “motivation influencing behavior through attitude” proposed by Kang et al. (2024), highlighting the specificity of the AI usage context. Technology attitudes often form prior to usage motivation. This phenomenon of “attitude preceding motivation” is relatively common in the adoption of emerging technologies. For instance, a user's attitude toward ChatGPT directly influences their exploratory usage motivation (Baek et al., 2024).

Second, the findings confirm that the three types of AI usage motivation, namely intrinsic motivation, identified regulation, and external regulation, fully mediate the relationship between AI attitude and AI-assisted creativity. The strength of these mediating effects exhibits a hierarchical pattern, with identified regulation being the strongest, followed by external regulation, and then intrinsic motivation. This result is consistent with the concept of attitude influencing behavioral intention from the Theory of Planned Behavior (Hill et al., 1977). This mechanism reflects the transition from affect to behavior; attitude itself does not directly predict creativity but indirectly influences it by activating specific motivations (Ruiz-Palomino and Zoghbi-Manrique-de-Lara, 2020).

Furthermore, the results concerning intrinsic motivation align with the views of Parwoto et al. (2024), who found that interest drives computer-based creativity. Although the high correlation between external regulation and creativity might seem contradictory to the traditional finding that “external rewards can undermine creativity” (Saether, 2020), this can be explained by considering the characteristics of AI technology. AI tools are inherently efficient and assistive, and external incentives might prompt students to more actively explore the practical functions of AI, thereby stimulating creative applications through high-frequency interaction.

Notably, the effect of identified regulation motivation was the strongest, corroborating the conclusion reached by Adler and Chen (2011) that collaborative creativity relies on identity-based motivation. As a highly internalized form of external motivation, it integrates autonomy and utility. Students recognize the value of AI for academic and career development, view it as a means to foster autonomous growth, and consequently form stable and enduring creativity. This finding also aligns with the observations of Ruiz-Palomino and Zoghbi-Manrique-de-Lara (2020), who noted that identified regulation motivation strengthens the mediating role in the attitude-creativity relationship.

This phenomenon might be related to the cognitive development stage of university students, who are in a critical period of value formation and place greater emphasis on the coexistence of autonomy and utility when evaluating tool value. On one hand, they need to actively explore to form their personal knowledge systems (an intrinsic need); on the other hand, external goals such as academic performance also influence their tool selection (an external need). Identified regulation effectively balances these two aspects of needs, making it more readily translated into sustained creative behavior. Meanwhile, although the proportion of intrinsic motivation's effect is the smallest, it remains a factor that cannot be overlooked, as long-term creative behavior often requires the support of intrinsic interest (Schmidhuber, 2010). It is also possible that purely interest-driven motivation is context-dependent, where as identified regulation is often more stable and aligns more easily with long-term learning goals (Ryan and Deci, 2000a,b).

Third, this study innovatively discovered that AI dependence has a differential moderating effect on the motivation-creativity relationship. Specifically, the inhibitory effect of AI dependence on intrinsic motivation validates the findings of Zhang and Xu (2025) regarding AI dependence leading to cognitive offloading. Furthermore, this result is consistent with the view of Ahmad et al. (2023) that when AI undertakes cognitive tasks such as information retrieval and solution generation, an individual's intrinsic motivation for autonomous exploration diminishes. When students frequently use AI to generate content, their brain's autonomous exploratory activities decrease, leading to a weakening of the intrinsically driven mechanism fueled by interest. The core of intrinsic motivation is the experience of autonomy regarding the task itself, whereas high AI dependence transforms the task into an operation of the tool, thereby reducing the individual's cognitive engagement in content generation. The enhancing trend of external regulation reflects the complementarity between instrumental goals and dependent behavior. The “performance reward expectation strengthening external motivation” discussed by Chen and Zhao (2025) is verified and extended here. High dependents tend to view AI as an efficient tool for achieving external goals, where the frequency of AI use forms a positive feedback loop with goal-achievement motivation, resulting in the phenomenon that higher dependence leads to a stronger effect of external incentives. Additionally, the stability of identified regulation suggests that internalized value cognition is not easily influenced by usage habits, indicating that when individuals perceive AI as an inherent need for academic development, their creativity is driven more by value than by the frequency of tool use.

### 5.1 Theoretical implications

This study focuses on university students' use of AI in learning tasks and their resulting creativity, an area where research has been relatively limited. According to this study, when university students actively use AI to complete learning tasks, they efficiently generate human-AI hybrid creativity, occurring in the form of AI-assisted creativity. This differs from the concept of AI-assisted creativity proposed by Mok et al. (2025) based on artistic creation. Grounded in human-AI collaboration, this study innovatively reveals the situation of university students utilizing AI to assist learning. It suggests that positive attitudes, intrinsic motivation, and identified regulation motivation toward using AI in learning may have broader influences and significance than previously thought. For instance, in the process of fostering student creativity development through AI-assisted learning, AI dependence can, to some extent, inhibit the intrinsic motivation for AI use. This study also shows that attitudes toward using AI extend the findings of Bewersdorff et al. (2025)—that positive AI attitudes can significantly predict intrinsic interest motivation—by demonstrating that positive AI attitudes more readily activate motivations related to value identification and external incentives, particularly identification with the value of AI in assisting task completion. The results support Zhang S. et al. (2024) view regarding the positive impact of external motivation on creativity, but further reveal that the role of identified regulation is more pronounced.

This phenomenon might be related to the particularity of the university learning context, where external factors such as academic competition, course requirements, and grade pressure tend to form associations with the instrumental value attributes of AI more naturally, whereas activating intrinsic interest is more challenging. Furthermore, the strongest influence of AI use attitude on identified regulation also corroborates this point: a positive use attitude is more easily translated into active identification with the value of AI technology, and this identification, in turn, most effectively promotes creative application, thereby enhancing learning outcomes.

By demonstrating how specific usage motivations, when students actively use AI for learning responsibly and appropriately, can inhibit AI dependence and promote creativity development, this study advances research on human-AI collaboration in higher education. It emphasizes the importance of positive attitudes toward AI technology use in learning tasks, guided by appropriate usage motivations, particularly highlighting the specific role of identified regulation motivation. This implies that within the higher education context, AI technology holds considerable practical value for university students' personal academic development, regardless of their initial attitude toward AI. This study expands our understanding of the internal mechanisms of student-AI collaboration and AI-assisted creativity development, especially in the scenario where students routinely use technological applications to complete learning tasks. This study validates the integration of the Theory of Planned Behavior and Self-Determination Theory in the context of human-AI collaborative creative behavior. It also demonstrates the inhibitory effect of intrinsic motivation on AI dependence and the significant exacerbating effect of external regulation motivation on AI dependence. Therefore, the model presented in this study provides theoretical support for enhancing personal creativity through intrinsic motivation and identified regulation when university students actively use AI in learning tasks, while also highlighting the differential impact of AI dependence on AI-assisted creativity across different types of AI usage motivation.

### 5.2 Practical implications

Educators and administrators in higher education should appropriately consider allowing students to use AI technology in learning tasks to enhance expected personal creativity. Although most university students hold positive attitudes toward using AI, especially for completing challenging learning tasks, the focus should be placed on how students can use AI correctly and responsibly. This involves placing greater emphasis on the intrinsic motivation for AI-assisted learning and avoiding excessive external motivation to prevent the formation of excessive AI dependence. Therefore, when students use AI technology, they should be emphasized the learning goals and motivations that are conducive to personal creativity development and long-term academic growth. Simultaneously, preventing them from developing excessive AI dependence is crucial. Higher education institutions need to formulate relevant educational policies and arrange training to help teachers effectively cultivate positive attitudes and reasonable motivations toward AI use among university students, thereby increasing their ability to develop problem-solving skills and creativity in learning tasks. Finally, it is necessary to monitor university students' AI dependence through assessment, surveys, and feedback mechanisms, to minimize the weakening of the student's personal component in AI-assisted creativity, while strengthening the cultivation of student AI literacy to enhance their motivation for using AI correctly, thereby more effectively fostering their personal creativity development.

### 5.3 Limitations and future research

This study elucidates the influencing mechanisms of creativity in university student-AI collaborative learning tasks, but several limitations remain. First, the sample selection did not account for disciplinary factors, including those from humanities, sciences, engineering, and arts, students from different majors might exhibit varying levels of AI dependence and demands for creativity. Second, as a cross-sectional study relying on a one-time questionnaire survey, it lacks data across different time dimensions, making it difficult to uncover dynamic relationships among variables. Finally, some potential variables were omitted from the model, such as AI literacy, learning task difficulty, and teacher guidance style, which could be factors influencing the relationship between AI attitude and creativity.

Future research could advance in the following directions. First, increase sample representativeness. Future studies should enhance the diversity of samples, encompassing both secondary and higher education levels and involving students from different disciplines. Second, employ longitudinal research methods to track dynamic relationships, observing the changes in AI attitudes, AI motivations, and AI-assisted creativity over time and examining their long-term impacts. Furthermore, the number of moderating and mediating variables included in the model was relatively limited. Future research could consider incorporating variables such as AI literacy, task types, and teacher intervention strategies to explore their roles in the “attitude-motivation-creativity” pathway.

## 6 Conclusion

University students demonstrate significant potential to enhance their creativity by utilizing AI technology to assist in learning tasks. Exploring the status of AI use among university students in Chinese higher education institutions, particularly in scenarios where AI assists in completing difficult learning tasks, and understanding how to use AI responsibly and appropriately are crucial. Therefore, this study investigated the mechanisms influencing the formation of AI-assisted creativity when university students use AI for learning tasks, through the lenses of AI use attitude, AI-assisted creativity, and the AI usage motivations of intrinsic motivation, identified regulation, and external regulation. Additionally, this study explored the moderating role of AI dependence in the relationships between these motivations and AI-assisted creativity. First, the results confirm a positive association between university students' AI use attitude and AI-assisted creativity. Second, the AI usage motivations—intrinsic motivation, identified regulation, and external regulation—positively mediate the relationship between AI use attitude and AI-assisted creativity. Third, AI dependence negatively moderates the relationship between intrinsic motivation for AI use and AI-assisted creativity. Fourth, AI dependence significantly moderates the relationships between both identified regulation and external regulation, and AI-assisted creativity. In learning tasks, assisting students with AI technology to solve difficult problems can promote the development of personal creativity. However, greater caution is needed during use. While actively utilizing AI technology, it is essential to strengthen the internality of AI usage motivation, cultivate responsible and correct AI use among students, prevent excessive AI dependence, and thereby increase their personal contribution within AI-assisted creativity.

## Data Availability

The raw data supporting the conclusions of this article will be made available by the authors, without undue reservation.
